# ITARA research programme: Investigating Integrated Tuberculosis And Respiratory care in Africa using transdisciplinary methods

**DOI:** 10.1136/bmjopen-2026-120068

**Published:** 2026-07-03

**Authors:** Jamilah Meghji, Nora Engel, Wanjiku Kagima, Idah Kinya, Yusufu Kionga, Maia Lesosky, Alphonce Liyoyo, Jason Madan, Stellah Mpagama, Joy Obase Ehrim, Adewale Ogundare, Obianuju Ozoh, Fred Orina, Joshua Parker Allen, Elizabeth Kendi Paul, James Potts, Lilian Tuwabunze, Nicola Yates, Jeremiah Chakaya Muhwa, on behalf of The ITARA Consortium, Jamilah Meghji

**Affiliations:** 1 National Heart and Lung Institute, Imperial College London, London, UK; 2 Athena Institute, Faculty of Science, Vrije Universiteit Amsterdam, Amsterdam, Netherlands; 3 Centre for Respiratory Diseases Research, Kenya Medical Research Institute, Nairobi, Kenya; 4 Kenyatta National Hospital, Nairobi, Kenya; 5 Strathmore University, Nairobi, Kenya; 6 Kibong’oto Infectious Diseases Hospital, Sanya Juu, Kilimanjaro, United Republic of Tanzania; 7 Centre for Health Economics, Warwick Medical School, Coventry, UK; 8 Department of Medicine, College of Medicine, University of Lagos, Lagos, Nigeria; 9 Department of Microbiology, University of Lagos, Lagos, Nigeria; 10 School of Public Health and Social Sciences, Muhimbili University of Health and Allied Sciences, Dar es Salaam, United Republic of Tanzania

**Keywords:** Tuberculosis, Pulmonary Disease, PUBLIC HEALTH, Health Services, HEALTH ECONOMICS, QUALITATIVE RESEARCH

## Abstract

**Abstract:**

**Introduction:**

Pulmonary tuberculosis (PTB) and chronic respiratory diseases (CRDs) are closely linked. Affected groups present with similar symptoms and share many risk factors (eg, poverty-related factors, smoking, occupational exposures). PTB is itself an independent risk factor for chronic lung disease. However, in many high TB-incidence settings health services for these conditions are provided separately, with little integration of prevention, diagnosis or care.

**Methods and analysis:**

We describe a transdisciplinary programme of research investigating strategies for integrated TB-CRD care in Arusha, Tanzania, Nairobi, Kenya and Lagos, Nigeria, using clinical, health economic, health systems and qualitative research methods. A prospective clinical cohort study will describe the burden and impact of non-TB respiratory disease (eg, asthma, chronic obstructive pulmonary disease, post-TB lung disease) among adolescents and adults presenting to primary and secondary health facilities with chronic cough who would normally be managed via TB care pathways. Health economics methods will explore patient costs of non-TB respiratory disease, facility-level costs of integrated TB/respiratory diagnostics and will develop a modelling framework to estimate the costs and consequences of integration more broadly. In-depth interviews, focus group discussions, observations and participatory methods will be used to explore lived experiences of chronic respiratory symptoms, disease and exposures among patients and providers, and to identify and address challenges around respiratory health and care. Lastly, existing TB and CRD healthcare services and systems in our three research sites will be described, and local, national and policy level understandings of ‘integration’ of TB and CRD care will be explored. Together, the findings of this work will be used to develop context-informed model(s) of integrated TB-CRD care and a theory of change and framework for evaluation in future implementation studies.

**Ethics and dissemination:**

Ethical approval has been obtained from Imperial College London in the UK, the Scientific Ethics Review Unit in Kenya, University of Lagos in Nigeria and the National Institute of Medical Research in Tanzania. Findings of this study will be presented in research publications and symposia, and will be shared with local communities and stakeholders.

**STRENGTHS AND LIMITATIONS OF THIS STUDY**

- Addresses a complex public health challenge—the integration of tuberculosis-chronic respiratory disease care—using transdisciplinary methods, with clinical, health economic, qualitative and health system substudies.
- Embeds community and stakeholder engagement as part of primary research activities.
- Collects data from three diverse urban sites in order to highlight context specific factors which are relevant to implementation and scale up of integrated TB-CRD care
- Generalisability limited by purposive selection of study facilities, within only three African cities.
- Although the work supports the development of robust model(s) of integrated care, it does not yet include pilot implementation work.

## Background

 The burden of tuberculosis (TB) and chronic respiratory disease (CRD) remains unacceptably high in low- and middle-income countries (LMICs), with 10.7 million incident cases of TB disease in 2024^1^ and respiratory diseases among the leading causes of morbidity and mortality globally.^2^ TB and CRDs are closely linked in LMICs. Both are conditions of deprivation, with shared risk factors including poverty, tobacco smoking and occupational exposures (eg, silica dust).^3 4^ Both are highly stigmatised and lead to long-term disability, health seeking and loss of income.^5 6^ TB is itself a direct cause of lung damage with up to half of pulmonary TB survivors left with residual post-TB lung disease (PTLD).^7^ People with TB and CRDs present with similar symptoms of cough and breathlessness and are largely defined as ‘people with presumptive TB (PPTB)’ and routed through TB services as an entry point to health services.

Despite this close relationship, TB and CRD care are delivered separately in LMICs. TB has historically been well managed via well-resourced National TB Programmes while public health programmes for the diagnosis and delivery of CRD care remain limited in many TB-endemic settings. The absence of integrated TB-respiratory care means that people with pulmonary TB who require respiratory support beyond anti-TB treatment may not receive it, while those with CRDs remain undiagnosed or are incorrectly treated with recurrent courses of antibiotics and TB medication for ongoing symptoms.^8^ Mistreatment of both of these conditions likely leads to adverse health outcomes with cost implications for both patients and the healthcare system.^9 10^

Despite growing awareness of the high burden of multimorbidity among TB affected communities, there remain few examples of coherent patient-centred services linking TB and non-communicable diseases in LMICs. We have little data on why, how and for whom these services might work. The lack of data is a significant barrier to implementation and scale up of integrated TB-CRD care.^11^ Global funding cuts have accelerated the need for integration of vertical care services (eg, HIV and TB care) and evidence on how to implement combined approaches to care in LMICs is much needed.

In this 4-year research programme (ITARA: Integrated TB And Respiratory care in Africa) we will use transdisciplinary research methods (clinical epidemiology, health systems, qualitative work, health economics) and work closely with communities and stakeholders to develop frameworks for the implementation and evaluation of integrated TB-CRD care in Africa, in order to address this evidence gap.

## Methods

### Research objectives and design

**Figure 1:**
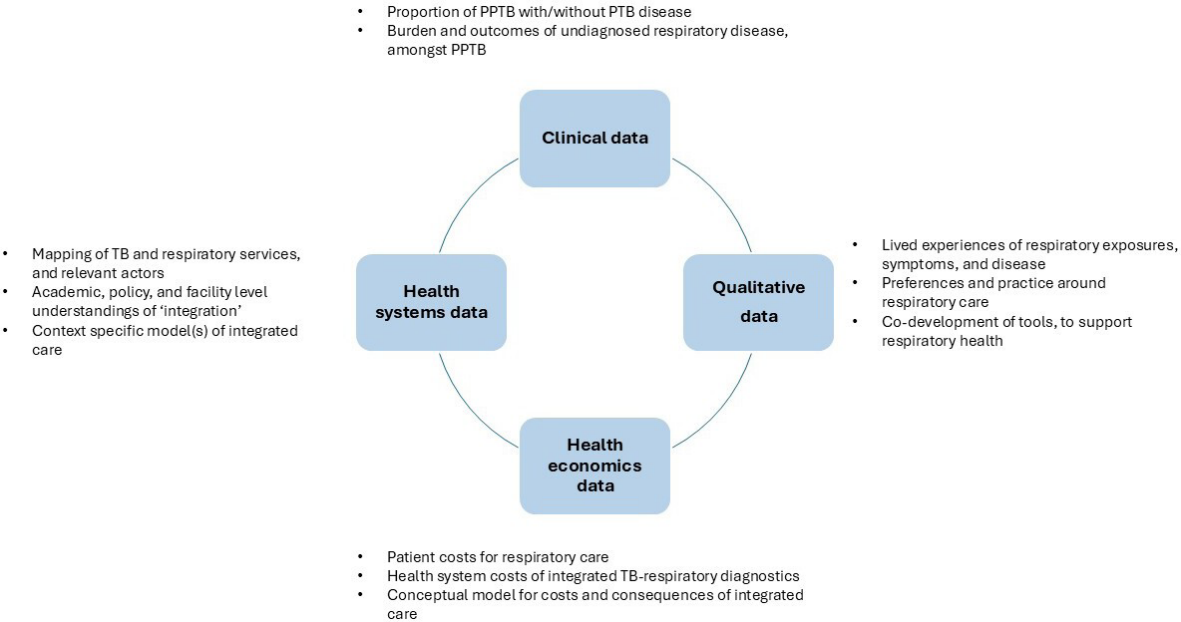
Interconnected methodologies and workstreams, within the ITARA Programme. ITARA, Integrated TB And Respiratory care in Africa; PPTB, presumptive TB; PTB, pulmonary TB; TB, tuberculosis.

ITARA is a transdisciplinary and observational programme of work, which will develop site appropriate model(s) of integrated TB-respiratory care, with an associated framework for impact/process evaluation, for future implementation and evaluation (figure 1).

### Study sites

Primary data collection is based in three urban African sites, purposively selected due to their diverse sizes and existing relationships between co-investigators: Arusha, Tanzania (population ∼600 000, national TB prevalence 172/100 000 in 2024),^1^ Nairobi in Kenya (population 4.8 million, local TB prevalence 806/100 000),^12^ and Lagos in Nigeria (population ∼17.2 million, national TB prevalence of 219/100 000).^1^ Research activities are focused around primary and secondary care health facilities at which individuals with respiratory symptoms may initially present, with two to four facilities purposively selected per city based on close proximity to each other, patient numbers, ability for patients to present directly with no need for referral, space and capacity for research activities, and the presence of TB services. Clinical and health economics studies collect primary data from these facilities only, while qualitative and health system studies collect data from associated referral and tertiary facilities, and community settings also. Recruitment to the workstreams started in May 2025, with data collection expected to continue to mid-2027.

### Clinical workstream

#### Study objectives

The clinical study aims to (1) estimate the proportion of adults and adolescents who present with cough ≥2 weeks, who do not have microbiological, radiological or clinically diagnosed TB disease, (2) estimate the proportion of these individuals with evidence of a non-TB CRD and (3) describe their clinical outcomes (mortality, incident TB treatment) and health seeking outcomes in the 6 months after presentation.

#### Study design

Participants are recruited into a baseline cross-sectional study, and complete study questionnaires. Those without microbiological evidence of TB disease progress into a prospective cohort study, with a 6-month follow-up period.

#### Recruitment

Adults and adolescents aged ≥15 years who self-present to outpatient services at participating health facilities on weekdays (Monday–Friday) are sequentially screened using the WHO four question TB symptom screen (any cough, fever, night sweats, weight loss), regardless of their primary reason for presentation. Those with any cough are defined as ‘people with presumptive TB’ (PPTB) and referred to the research team for formal screening using study inclusion and exclusion criteria (table 1). In order to enrich the study for those with chronic rather than acute conditions, only PPTB with cough ≥2 weeks are enrolled. Adolescents under 18 years are eligible if they provide assent and if a guardian is available to provide informed consent, in keeping with national guidelines in each country. Pregnant patients are excluded because of the requirement for chest X-ray (CXR) imaging and spirometry within the study.

**Table 1:** Inclusion/exclusion criteria for the baseline cross-sectional study

Inclusion criteria	Exclusion criteria
Self-presenting for care. Positive WHO TB symptom screen. Aged ≥15 years. Chronic cough ≥2 weeks. Written informed consent (or assent) for study. Live or work locally to health facility.	Already on TB treatment. Acutely unwell or requiring hospital admission. Identified via active case finding, or referred from another health facility or outpatient clinic. Pregnant.

TB, tuberculosis.

#### Data collection

All study participants complete questionnaires on the day of presentation and recruitment, to collect data on demographics, current TB and respiratory symptoms, health seeking over the past 6 months, medical history including previous TB disease, respiratory and occupational exposures, socio-economic situation and health related quality of life using the EQ-5D-5L score (online supplemental table S1). Individuals who report HIV negative or unknown status are offered repeat HIV testing. All participants are referred for TB diagnostic tests as per local guidelines, including a single spontaneous sputum sample for Xpert MTB/Rif Ultra, and the urinary LAM test (Lateral Flow Urine Lipoarabinomannan Assay) for people living with HIV in Tanzania.

Those who are Sputum Xpert MTB/Rif Ultra negative or who are unable to produce sputum, and who have a negative urinary LAM test (if completed) are eligible to continue into the cohort study. Participants are sequentially recruited into the cohort study, with weekly maximum numbers set per site (n=15–20), in order to allow for completion of study procedures. Participants who are unable to return to the facility for further investigations within the study window (4 days from presentation) are excluded.

Cohort study participants complete clinical observations, blood tests (full blood count (FBC), CD4 count if HIV positive, stored serum sample), a nasopharyngeal swab (stored in viral transport medium) and are asked to submit two spontaneous sputum samples (for mycobacteria growth indicator tube (MGIT) culture and stored sample) within 4 days of their initial presentation. Digital posteroanterior CXR imaging and pre/post bronchodilator spirometry are completed within 7 days of baseline presentation.

Cohort study participants are contacted by phone at 8 weeks to collect data on their current symptoms, and health seeking since recruitment, including whether or not they have been started on TB treatment. TB MGIT culture results are shared with participants at this time—those who are culture positive are linked to care and exited from the cohort. Participants who remain in the study complete a final in-person study visit at 26 weeks. This includes repeat questionnaires (current symptoms, further health seeking, TB treatment initiation, EQ-5D-5L), clinical observations, sputum samples for storage and MGIT culture, and CXR and pre/post bronchodilator spirometry. Final MGIT culture results are communicated to participants by phone 8 weeks later. The TB register is reviewed for participants who are started on TB treatment during cohort follow-up, and TB diagnosis/treatment data extracted. Next of kin are contacted for individuals who are lost-to-follow-up, in order to determine vital status.

#### Linkage to care

Investigation results (clinical observations, HIV test, FBC, CD4 count, CXR images, spirometry results) are reviewed by a member of the study team in real-time, and participants with abnormal results are linked to healthcare providers at the participating health facilities. Decision making about further investigations, treatment and care remains with local clinical providers, as per the standard of care and no direct clinical care is provided by the study team. A series of CXR training sessions was delivered to local facility staff at the start of the study, with further training on local CRD guidelines and referral pathways over the course of the study, to support local clinical care.

#### Sample size

A total sample size of 1200 study participants, with ∼400 from each participating country was chosen for the cohort study, in order to allow us to estimate the prevalence of key CRDs (asthma, chronic obstructive pulmonary disease (COPD) and PTLD) with sufficient precision. Key CRDs were assumed to have a prevalence between 10% and 20%, providing at least 5% precision to estimate a 95% CI. Based on previous studies, we estimated that between 30% and 50% of PPTB would be Xpert Ultra positive, requiring at least 800 PPTB recruited into the cross-sectional study per country, in order to meet the cohort study target.

#### Data interpretation

Quality control of spirometry testing and regular feedback/retraining of technicians in study sites is provided throughout the cohort study. Final spirometry reads will be completed by an external expert using the race-neutral Global Lung Function Initiative reference ranges. All CXR images are reviewed in real time by a member of the local study team (doctor or radiologist), and shared with local clinical providers, but will also be formally reported by a trained senior radiologist post hoc using a standardised proforma, for research purposes. A prespecified data-driven algorithm will be developed to use clinical, blood test, spirometry and imaging data from both baseline and 6-month time points, to allocate cohort study participants to a single dominant respiratory diagnosis. A clinical multidisciplinary team meeting will be used to allocate a diagnosis to those who do not meet fixed criteria or where there is uncertainty. Diagnostic criteria will focus on conditions which we hypothesise will be most common including active TB disease, upper/lower respiratory tract infections, PTLD, COPD and asthma.

#### Data analysis

In the cross-sectional study, the proportion of individuals with microbiological, imaging or clinically diagnosed TB will be estimated with 95% CIs and participant characteristics described overall and by TB diagnosis. Associations will be estimated using appropriate generalised linear regression models, adjusted for covariates and confounders which will be identified by mapping to a direct acyclic graph. Among cohort study participants, respiratory diagnosis and clinical outcomes will be described and factors associated with or predictive of clinical outcomes will be estimated using multivariable regression modelling approaches. Primary analyses will be conducted using merged data, from all health facilities and all study sites, with adjustment for study site. Missing data will be reported, and multiple imputation considered for low percentage (<5%) missing covariates or confounders. Stratified analyses by study site will also be presented, and pooled prevalence estimates for key diagnoses across sites will be estimated using individual participant meta-analysis approaches. Variables included in the analyses cover all aspects of the data collection including participant demographics, HIV and previous TB status, self-reported respiratory symptoms and exposures, clinical measurements, health seeking behaviours and costs incurred.

### Health economics workstream

#### Study objectives and design

The health economics component of ITARA asks what investment would be required to integrate TB and CRD in routine care, and describes the potential impact of this on patients and health systems. Specific objectives include to (1) estimate the direct and indirect costs incurred by PPTB and their families who do not have confirmed TB, and explore predictors of high and catastrophic costs in this group, (2) estimate the costs to health systems of implementing the enhanced diagnostic pathway for CRDs, which is being employed for PPTB in the ITARA clinical study above, and (3) develop a conceptual model of the mechanisms through which different integration strategies would affect costs and consequences for patients with CRD and health services.

#### Patient costing study

This study is nested within the ITARA clinical study, where both baseline and 6-month questionnaires include a patient cost section adapted from the WHO TB Patient Cost Survey.^13^ Key domains of economic data will be collected from participants at these time points including: direct medical costs (eg, consultation fees, medications, diagnostic tests, hospitalisation); direct non-medical costs (eg, transportation, accommodation, food expenses during care-seeking); indirect costs (eg, lost income due to time lost while ill or seeking care, caregiver time); and coping mechanisms (eg, borrowing, asset sales, reduced household consumption). Data analysis approaches will be consistent with the clinical study, described above. Results will be presented on mean and median costs, the prevalence of catastrophic costs and the frequency and magnitude of coping mechanisms. Generalised linear multivariate regression models will explore the association of these quantities with presence or absence of a TB or CRD diagnosis, and socio-economic and sociodemographic indicators such as age, sex, occupation, HIV status and self-reported history of TB disease.

#### Health system costing study

This study will be completed at two primary healthcare facilities in Kenya, from which the clinical study is recruiting. A micro-costing exercise will be used to evaluate the costs of the enhanced PPTB diagnostic pathway implemented by the ITARA clinical study from a health system perspective. It will include human resource, test unit and operational costs for the additional respiratory diagnostics included in the clinical study (clinical observations, blood tests, spirometry and CXR), and will be completed in three stages. First, we will identify the resources necessary for the implementation of this diagnostic pathway based on standard operating procedures and direct observation, and will classify these resources into recurrent costs (staff, test kits and consumables, protective equipment and variable overheads) and capital costs (fix overheads, building and equipment). Second, we will measure the consumption of the identified resources using facility-level records, observation, interviews and time-motion studies. Finally, the costs of resource consumption will be valued using both a bottom-up^14 15^ and a top-down approach,^16 17^ and reasons for discrepancies (eg, capacity utilisation) will be identified.^18^

#### Modelling study

The ITARA clinical study and the provider costing study above are focused on integrated diagnostics for TB and CRD. In order to support future economic evaluation of broader integration across the TB and CRD care pathways, a conceptual model for estimating the costs and consequences of integration will be developed. This work will be completed in line with the recommendations of the ISPOR-sMDM Modelling Good Research Practices Task Force,^19^ and in collaboration with the health systems substudy below. The model will provide a diagrammatic and textual representation of the CRD diagnostic and treatment pathway, the components of integration and the causal relationships linking service integration with costs and consequences relevant to patients, providers and society more broadly. The conceptual model will align with and extend the integration framework developed by the health systems workstreams by providing the information needed for researchers and policymakers to develop decision-analytical simulation models, predicting the costs and consequences of implementing specific integration initiatives.

### Qualitative workstream

#### Study objectives

This work will use a transdisciplinary approach to explore the lived experience of individuals, communities and providers in urban settings around respiratory health, and will co-develop tools to support CRD-care. Specific objectives include to (1) understand how community members, patients and providers perceive and experience respiratory exposures, symptoms and disease, (2) describe existing approaches to and preferences for respiratory care among these groups, (3) identify critical challenges around respiratory health, and to co-develop tools to address these challenges. Initial work will be based in Arusha, Tanzania, before adaptation across sites. A human-centred design approach involving principles like empathy for users, iterative development and collaboration will be used throughout.^20^

#### Stakeholder mapping and engagement

This work will map and engage key stakeholder groups with experiential knowledge of CRDs separately through in-depth interviews (estimated n=70), focus group discussions (estimated n=30) and observation of clinical consultations, health seeking journeys, participants who are living with environmental exposures, and interactions between stakeholders. Participants will include people with respiratory symptoms who do/do not attend healthcare services, TB-survivors, people in occupations with significant exposures to dusts and fumes (eg, motorcycle taxi drivers and waste pickers exposed to exhaust fumes, those working in mining, maize grinding or charcoal selling), facility and community-based healthcare workers, informal and formal care providers, and community leaders. Argumentation trees will be developed for each stakeholder group to structure and visualise perspectives and experiences about chronic respiratory symptoms, exposures and diseases.^21^

#### Problem framing and co-design

Stakeholders who showed interest and have relevant diverse backgrounds and experience (eg, across gender, occupation, community roles) will be invited to participate in problem-structuring workshops.^22^ Here, the structured learning dialogue technique will be used to share initial qualitative data, and preliminary data from the clinical study with participants, and to support them to reflect on their own and others’ perspectives and experiences, in order to develop a shared problem definition around respiratory health. Tools and approaches which could address this problem will be explored using the PRODUCES framework.^23^ The nature of these tools is not prespecified, but will be developed in response to the challenges identified by participants, and will be iteratively revisited during the project. The co-design pathways technique will be used to run workshops where participants will cocreate the tools suggested, together with an evaluation framework which would allow the monitoring of resources used to implement these tools, and identification of (un)desired consequences for different stakeholders.

#### Adaptation across sites

In the final stage of the work, stakeholder workshops will be held in Nairobi and Lagos, to share findings and tools from Tanzania and consider the applicability of these tools to these different urban settings, and to consider if/how these tools might be adapted to the local context.

### Health systems workstream

#### Study objectives

The health systems work aims to understand how TB and CRD services are organised, governed and delivered in each of the research sites, and to develop a contextually grounded framework for their potential integration. Specific objectives include to (1) identify the services available for TB and CRD care within each site, key actors within them and relationships between actors and services, and (2) explore perspectives on the meaning of the term ‘Integration’, from the academic literature, policy and guidelines, and the perspectives of stakeholders directly involved in the delivery of TB-CRD care, (3) co-develop a conceptual framework for integration, which considers both the form and principles of integration, across TB and CRD care pathways.

#### Study design

The study will use semistructured interviews and observations of patients and providers within primary and secondary care facilities linked to the ITARA clinical study, as well as broader services to which they are connected including public, private, voluntary and community organisations, and tertiary referral centres. Desk-based reviews of policies, guidelines, budgets and governance structures will be used to triangulate data, and broader networks of local, regional, national and international stakeholders will be engaged. The work will run across the three study sites in Arusha, Nairobi and Lagos.

#### Health system mapping

Holistic ‘maps’ describing the nature and organisation of the TB and CRD health systems will be developed. These will describe the various services providing TB or CRD care around the ITARA clinical study sites, using the expanded WHO Health System framework (governance, financing, service delivery, knowledge infrastructures, spaces and supplies, human resources and communities).^24 25^ An actor analysis will be completed to identify key individuals involved in TB and CRD care, and their roles, understandings and relationships. Links between organisations and individuals—both formal and informal—will be described.

#### Meanings of ‘integration’

This work investigates both the multiple understandings of the concept of ‘integration’ held by academics, policymakers, healthcare providers and other stakeholders. Data will be collected through reviews of academic literature and policy documents, and in-depth interviews with key stakeholders, to explore how this concept has been framed over time, the key agendas driving this and understandings of this term. Empirical data from healthcare workers will be used to obtain a ‘bottom-up’ understanding of how this concept is understood on the ground, in our study sites.

#### Implementation framework for integration

Workshops and in-depth interviews with stakeholders—both within and outside of the ITARA research team—will be used to develop an initial framework for integration, which considers both the form of integration, and the key principles underlying this. This framework will run across the TB-care and CRD-care pathways and will be iteratively refined through interviews and discussions with stakeholders—a broad range of local, regional and international participants will be included to ensure that proposed models of integration are locally grounded, adaptable and feasible in practice. These engagements will focus on the perceived benefits, barriers and trade-offs of integration, as well as operational feasibility and financial sustainability. Comparative analysis across the three countries will highlight both commonalities and context-specific challenges.

### Patient and public involvement

Stakeholder engagement is a critical component of the primary research activities above. Alongside this we will complete stakeholder mapping in each study country, reflect on the roles of each group within our research activities (Inform, Consult, Involve, Collaborate, Empower),^26^ and will develop a structured plan of engagement at the local, regional, national or international level to facilitate this. Community members—defined as members of the local population who are affected by TB or CRDs, and engage with local health services—are considered a priority stakeholder group, and community advisory boards will be established in each site to inform research activities over the course of the work.

### Shared outputs

Data and activities within the clinical, health economics, qualitative and health systems workstreams are closely linked, and will be brought together in workshops with the research team/critical stakeholders. We will use these data to co-develop (1) context specific model(s) of integrated TB-CRD care, which operate across the TB and CRD clinical care pathways, (2) a theory of change describing how and for whom these models might work, and (3) an evaluation framework which captures both positive and negative impacts of this novel approach to care. These outputs will inform the design of an implementation study to examine the impact of an integrated TB-CRD care.

## Ethics and dissemination

Ethical approval has been obtained from Imperial College London in the UK (Ref 7286073, study sponsor), the Scientific Ethics Review Unit in Kenya (Ref 117/5143), University of Lagos in Nigeria (ADM/DSCST/HREC/APP/7062) and the National Institute of Medical Research in Tanzania (Ref 4876). Data is being curated at Imperial College London, and anonymised quantitative data will be made available on request with publications. Research dissemination will be through academic publications, conferences and dedicated meetings with stakeholders and community groups.

## Discussion

This programme of work uses transdisciplinary methods to explore strategies for integrated TB and respiratory care in primary and secondary healthcare facilities at three diverse urban sites in Africa. Together, these studies will describe the clinical need, potential structure, likely cost implications and patient and provider perspectives around integrated TB-respiratory care. It will inform the development of context-appropriate model(s) of integrated care, patient centred tools to support respiratory health, a theory of change and clear evaluation framework for use in further pilot and implementation studies. We acknowledge that our data will be drawn from a limited number of urban sites, and small number of health facilities and regions in Africa only, but hope that the heterogeneity within this sample will allow us to identify areas of similarity and difference, and will help us to understand if and how context could shape any future models of care.

## Supplementary material

10.1136/bmjopen-2026-120068 online supplemental file 1
