# Supplementary material for: ITARA research programme: Investigating Integrated Tuberculosis And Respiratory care in Africa using transdisciplinary methods
Source: BMJ Open. 2026 Jul 3;16(7):e120068. doi: 10.1136/bmjopen-2026-120068 (PMC13343057; doi:10.1136/bmjopen-2026-120068)
Supplement: online supplemental file 1 [file bmjopen-16-7-s001.docx]

**Supplementary materials**

**The ITARA research programme: investigating Integrated Tuberculosis and Respiratory care in Africa using transdisciplinary methods**

Jamilah Meghji, Nora Engel, Wanjiku Kagima, Idah Kinya, Yusufu Kionga, Maia Lesosky, Jason Madan, Stellah Mpagama, Joy Obase Ehrim, Adewale Ogundare, Obianuju Ozoh, Fred Orina, Joshua Parker Allen, Elizabeth Kendi Paul, James Potts, Lilian Tuwabunze, Nicola Yates, Jeremiah Chakaya Muhwa, on behalf of The ITARA Consortium

Table S1: Clinical study procedures

| Participant group | Tool/sample | Investigation |
| --- | --- | --- |
| Cross-sectional study | Study Questionnaire | Demographics  Socioeconomic position  Medical history, including previous TB disease / treatment  General health & respiratory symptoms  Health seeking, and direct / indirect costs incurred  Respiratory and occupational exposure questionnaire  CRD, COPD and Asthma screening questionnaires  Quality of life questionnaire (EQ5D5L)  Next of kin contact details |
|  | Facility investigations | HIV testing  XPert MTB/Rif  Urinary LAM* |
| Cohort study – Baseline | Imaging | Chest radiograph |
|  | Clinical assessment | Clinical observations - HR, BP, RR, Oxygen saturation, BMI  Pre/post bronchodilator spirometry |
|  | Sampling | Full blood count  CD4 count, if HIV positive  Sputum sample for MGIT culture  Stored nasopharyngeal swab  Stored sputum sample  Stored serum sample |
| Cohort study –  8 weeks | Questionnaire | General health  TB treatment history  Health seeking episodes |
| Cohort study –  26 weeks | Questionnaire & Notes review | General health & respiratory symptoms  TB treatment history  Health seeking, and direct / indirect costs incurred  Quality of life questionnaire (EQ5D5L) |
|  | Imaging | Chest radiograph |
|  | Clinical assessment | Clinical observations - HR, BP, RR, Oxygen saturation, BMI  Pre/post bronchodilator spirometry |
|  | Sampling | Sputum sample for MGIT culture  Stored sputum sample |
|  | TB register review | TB treatment outcome |

*Use as per local guidelines
