# Supplementary material for: ITARA research programme: Investigating Integrated Tuberculosis And Respiratory care in Africa using transdisciplinary methods
Source: BMJ Open. 2026 Jul 3;16(7):e120068. doi: 10.1136/bmjopen-2026-120068 (PMC13343057; doi:10.1136/bmjopen-2026-120068)
Supplement: Supplementary file 2 [file bmjopen-2026-120068.reviewer_comments.pdf]

PEER REVIEW HISTORY

BMJ Open publishes all reviews undertaken for accepted manuscripts. Reviewers are asked to complete a checklist review form and are provided with free text boxes to elaborate on their assessment. These free text comments are reproduced below.

ARTICLE DETAILS

Title (Provisional)

The ITARA research programme: Investigating Integrated Tuberculosis and Respiratory care in Africa using transdisciplinary methods

Authors

Meghji, Jamilah; Engel, Nora; Kagima, Wanjiku; Kinya, Idah; Kionga, Yusufu; Lesosky, Maia; Liyoyo, Alphonce; Madan, Jason; Mpagama, Stella G; Obasi-Ehrim, Joy; Ogundare, Adewale; Ozoh, Obianuju B; Orina, Fred; Parker-Allen, Joshua; Paul, Elizabeth Kendi; Potts, James; Tuwabunze, Lilian; Yates, Nicola; Chakaya, Jeremiah; The ITARA Consortium, N/a

---

This article was not externally reviewed at BMJ Open. Protocols that have been independently assessed prior to submission to BMJ Open are usually fast-tracked to publication on the grounds that further substantial changes will not be possible. This independent assessment will usually be external, independent review for both a substantial grant award from a non-commercial or government funder and ethics approval.
